# Usage of Plant Food Supplements (PFS) for weight control in six European countries: results from the PlantLIBRA PFS Consumer Survey 2011-2012

**DOI:** 10.1186/s12906-016-1227-5

**Published:** 2016-07-28

**Authors:** Alicia Garcia-Alvarez, Raimon Mila-Villarroel, Lourdes Ribas-Barba, Bernadette Egan, Mihaela Badea, Franco M. Maggi, Maija Salmenhaara, Patrizia Restani, Lluis Serra-Majem, on behalf of the PlantLIBRA PFS Consumer Survey group

**Affiliations:** 1 Fundación para la Investigación Nutricional, Parc Científic de Barcelona, Baldiri i Reixac, 4-8, Barcelona, 08028 Spain; 2 Food, Consumer Behaviour and Health Research Centre, University of Surrey, Guildford, Surrey GU2 7XH UK; 3 Department of Fundamental, Prophylactic and Clinic Specialties, Faculty of Medicine, Transilvania University of Brasov, Bdul Eroilor Nr 29, Brasov, 500039 Romania; 4 Dipartimento di Scienze Farmacologiche e Biomolecolari, Università degli Studi di Milano, via Balzaretti 9, Milano, 20133 Italy; 5 Finnish Food Safety Authority Evira, Mustialankatu 3, Helsinki, 00790 Finland; 6 Ciber Obn Fisiopatología de la Obesidad y la Nutrición, Instituto de Salud Carlos III, C/Monforte de Lemos 3-5, Pabellón 11, Planta 0, Madrid, 28029 Spain; 7 Institute of Biomedical and Health Research of the University of Las Palmas de Gran Canaria, Campus Universitario de San Cristobal, Paseo de Blas Cabrera Felipe, Las Palmas de Gran Canaria, 35016 Spain

**Keywords:** Weight control, Body mass index, PlantLIBRA survey, Plant food supplements, European

## Abstract

**Background:**

Obesity is increasing worldwide and weight-control strategies, including the consumption of plant food supplements (PFS), are proliferating. This article identifies the herbal ingredients in PFS consumed for weight control and by overweight/obese dieters in six European countries, and explores the relationship between their consumption and their self-reported BMI.

**Methods:**

Data used were a subset from the PlantLIBRA PFS Consumer Survey 2011-2012, a retrospective survey of 2359 PFS consumers. The survey used a bespoke frequency-of-PFS-usage questionnaire. Analyses were performed in two consumer subsamples of 1) respondents taking the products for “body weight reasons”, and 2) “dieters for overweight/obesity”, to identify the herbal ingredients consumed for these reasons. The relationship between the 5 most consumed herbal ingredients and self-reported BMI in groups 1 and 2 is explored by comparing BMI proportions of consumers vs. non-consumers (using Chi-squared test).

**Results:**

252 PFS (8.8 %) were consumed for “body weight reasons” (by 240 PFS consumers); 112 PFS consumers (4.8 %) were “dieting for overweight/obesity”. Spain is the country where consuming herbal ingredients for body weight control and dieting were most popular. Artichoke was the most consumed herbal ingredient. Considering only the 5 top products consumed by those who responded “body weight”, when using the total survey sample, a greater proportion of BMI ≥ 25 was observed among consumers of PFS containing artichoke and green tea as compared to non-consumers (58.4 % vs. 49.1 % and 63.2 % vs. 49.7 % respectively). Considering only the 5 top products consumed by “dieters” and using only the “dieters” sample, a lower proportion of BMI ≥ 25 was observed among pineapple-containing PFS consumers (38.5 % vs. 81.5 %); however, when using the entire survey sample, a greater proportion of BMI ≥ 25 was observed among artichoke-containing PFS consumers (58.4 % vs. 49.1 %).

**Conclusions:**

A comparison of results among the scarce publications evaluating the use of weight-loss supplements at the population level is limited. Nevertheless every hint is important in finding out which are the self-treatment strategies used by overweight/obese individuals in European countries. Although limited by a small sample size, our study represents a first attempt at analysing such data in six EU countries. Our findings should encourage the conduction of further studies on this topic, long-term and large sample-sized studies, ideally conducted in the general population.

## Background

Obesity is a global epidemic [1, 2] and consequently many individuals are seeking strategies to reduce their body weight and fat levels. These strategies may include weight-loss food supplements, including plant food supplements (PFS), such as appetite suppressants or those increasing resting metabolism [3].

PFS that claim to contribute to weight loss are marketed worldwide and readily available over the Internet [4–6]. This increased usage has coincided with a resurgence of interest in nutritional therapy and complementary and alternative medicine (CAM) [7]; PFS and dietary therapies for weight loss are among the most common CAM modalities [8]. Various reasons underlie this preference: the therapies are promoted as requiring less effort than other behavioural changes (i.e. diet and exercise); are heavily advertised with claims of effectiveness; are readily available without a prescription [9]; are commonly marketed on the Internet [10]; are believed to be “natural” and “harmless”; and, at least in the EC countries, are regulated as foods rather than medicines [11]. Moreover, there is no perceived need for professional assistance with these strategies and individuals who cannot afford to visit a physician often view PFS as a more accessible solution [12]. For many other individuals, these strategies represent alternatives to failed attempts at losing weight using more conventional approaches; these consumers are likely to combine strategies or use these supplements at doses higher than recommended [9, 12].

The fact that PFS used for weight loss do not require rigorous safety controls before entering the market is causing a serious public health problem, evidenced by the increasing number of studies of hepatotoxicity from their use worldwide [13–17]. However, these studies have major methodological limitations that make it difficult to evaluate causality [17].

Actions are already been taken to tackle this problem in countries with the highest consumption of weight-loss supplements, such as the United States (US) or Japan [18]. In the US, the National Institutes of Health (NIH) committed substantial funding to dietary supplement research in the financial years 2009–2011 with the objective of expanding the scientific knowledge base on the efficacy and safety of dietary supplements, with botanicals being the dietary supplement ingredients receiving the most funding [19]. In Europe, the assessment of the efficacy and safety of food supplements including herbal ingredients is also being addressed, driven by the increasing usage of these products [20–22].

The literature on weight-loss PFS and their individual ingredients is extensive and includes reviews [17], randomized controlled trials (RCTs) assessing the effectiveness of these products [12, 23–26] or of individual herbal ingredients (such as *Phaseolus vulgaris* [27]. Other available research includes RCTs that assess the effectiveness/efficacy of individual herbal ingredients for weight loss [28, 29] and their adverse effects [22, 23]; finally, other research has evaluated the availability of weight-loss products (including herbals/botanicals) in the local markets [30, 31].

However, very few surveys have addressed the use of these particular products by consumers, with limited information on who is using them and which herbal ingredients are included in the weight-loss PFS reported by actual users. A number of multi-country, national, regional or local surveillance surveys have asked about the use of supplements [32–36], with some including sections on CAM and herbal supplements [37–42]. In spite of this, the focus is not specifically on weight-loss herbal supplements, but rather any supplement use, such as vitamin and mineral use, or CAM use or the use of the most commonly taken herbs to treat a specific health condition [41]. At the European level, the recent PlantLIBRA PFS Consumer Survey [43] is the first source of user data available that has allowed an analysis on weight-loss PFS in 6 European countries, and whose results are presented in the current paper.

Few studies on the use of herbal ingredients for weight-loss have been identified, with the larger-scale ones being conducted in the US. The most directly related one used data from the 2002 National Physical Activity and Weight Loss Survey (final *n* = 9,403); it assessed the prevalence and duration of non-prescription weight-loss supplement use (8.7 % had past year use, and use by adults with obesity was substantially higher than that of normal-weight individuals), the associated weight-control behaviours, the discussion of supplement use with a health care professional, and specific ingredient use (73.8 % used supplements containing a stimulant including ephedra, caffeine, and/or bitter orange) [44]. Another US study used data on CAM use from the 2002 National Health Interview Survey (NHIS) Alternative Medicine Supplement (*n* = 31,044) and compared the use of CAM overall, within the previous year, between four categories of adult BMI [45]. A third and smaller US study used data from a 2005–2006 nationally representative survey (*n* = 3,500 adults), and assessed dietary supplement use for weight loss and perceptions of safety, efficacy and regulatory oversight of these products [9]. Outside the US, a 2009 survey in the Polish city of Szczecin evaluated the range of weight-loss programmes and behaviours associated with the use of slimming supplements (appetite inhibitors or fat burning and thermogenesis enhancers), observed among 300 female university students [46]. The most recent study was a cross-sectional population-based survey conducted in 2,732 adults living in the Brazilian city of Pelotas that aimed to determine the prevalence of weight-loss practices and use of substances for weight-loss during the 12 months preceding the interview [47].

Because weight-loss PFS usage data are very scarce, with almost no data on the actual herbal ingredients consumed, the objectives of this paper are two-fold: 1) to identify the PFS herbal ingredients consumed for weight loss and/or control in 6 European countries, and 2) to explore the relationship between the consumption of these herbal ingredients and the self-reported BMI of their consumers. A subset of data from the six-European-country “PlantLIBRA PFS Consumer Survey 2011–2012” has been used.

## Methods

### Survey sample

This study was carried out within the PlantLIBRA project (FP7-EC funded project n°245199). Data on PFS usage were collected in Finland, Germany, Italy, Romania, Spain and the United Kingdom, in a cross-sectional, retrospective survey of 2359 PFS consumers, using a bespoke frequency-of-PFS-usage questionnaire. Further details of the methodology of the survey (sampling, questionnaires, data collection, databases, etc), and the concepts and definitions used, can be found in Garcia-Alvarez et al. [43].

### Study samples

Analyses were performed on 3 subsamples of the PlantLIBRA PFS Consumer Survey population: 1) PFS consumers who responded to be taking the products for “body weight reasons” (*n* = 240), when asked *“For what reason(s)/condition(s) did you take this product?”,* 2) PFS consumers who reported to be “dieting for overweight/obesity” (*n* = 112), when asked *“Please indicate the special diet that you follow”,* and 3) PFS consumers who belonged to both subsamples 1 and 2 (*n* = 67), i.e. who responded to take the product for “body weight reasons” while “dieting for overweight/obesity”.

### Variables

A number of variables were created and/or recoded in the original data set [20] to facilitate reporting and analysis, including: 1) “body weight reason”, with two categories: “Responded body weight” (products taken for “body weight”) and “Did not respond body weight” (products not taken for “body weight”); 2) “dieting”, with two categories: “dieting for overweight/obesity” (consumers dieting for overweight/obesity) and “not dieting for overweight/obesity” (consumers not dieting for overweight/obesity); 3) “BMI”, calculated from self-reported weight and height, and for which WHO criteria [48] were used to categorise individuals as “underweight-and-normal weight” (BMI < 25 kg/m^2^) and “overweight-and-obese” (BMI ≥ 25 kg/m^2^); 4) “education level”, defined as low, medium, and high; 5) “employment status”, defined as “currently employed” and “other groups”; 6) “physical activity”, calculated using the short version of the IPAQ [49] and defined as low, moderate or high; 7) “food frequency”, defined as times/day of fruit, vegetables, bakery and pastries, soft drinks and fast food.

### Statistical analyses

The statistical package SPSS for Windows v. 18 (IBM Corporation, Somers, NY, USA) was used for data analysis.

The subsamples of respondents and non-respondents using PFS for body weight reasons were described in terms of the above variables/characteristics, using both Chi-squared and *t* tests for categorical and mean comparisons (*p* < 0.05 for significance). Frequencies and percentages for the variables “responded body weight reason” and “dieting for overweight/obesity” were stratified by country. Absolute frequencies, percentages and 95 % confidence intervals for the top 20 herbal ingredients in products taken by respondents and non-respondents of body weight reasons and by overweight/obesity dieters and non-dieters were calculated, as well as those for the top 10 herbal ingredients in products taken by consumers who responded body weight and who were simultaneously dieting for overweight/obesity. For the purpose of this paper, the frequency of an individual herbal ingredient was defined as “the number of times that herbal ingredient was found as ingredient in the total pooled number of PFS consumed by all respondents of the subsamples”. Moreover, each herbal ingredient only counted once regardless of the number of times it was consumed by a respondent. Finally, it was not taken into account if the herbal ingredient came from a single- or multi-ingredient product, i.e. no “weight” was given to the particular herbal ingredient within the product.

Chi-squared tests were used to test the relationship between the 5 most consumed herbal ingredients and self-reported BMI in subsamples 1 and 2, by comparing BMI proportions of consumers vs. non-consumers of these herbal ingredients (*p* < 0.05 for significance). Comparisons were made using a) subsample 1 and subsample 2 in which the top 5 consumed herbal ingredients were actually identified (*n* = 240 and *n* = 112 respectively), and b) the total survey PFS consumer sample (*N* = 2359), in order to increase the power of the test. Finally, absolute frequencies of the top herbal ingredient contained in the consumed PFS were stratified by country.

It is important to bear in mind that when reporting the results, the unit of analysis varies depending on the variables used, i.e. for certain variables the unit is an individual respondent, for others it may change to the PFS product level, or to the level of the herbal ingredient contained in the product. Furthermore, data were not weighted by the population size because of the study methodology selected, whereby all country samples were very similar in size and included only PFS consumers. All results presented in the tables represent analysis of the raw data.

## Results

### Characteristics of PFS users for reasons of body weight and of PFS users for other reasons

Table 1 shows the characteristics of the overall survey sample, and also of the subsample of consumers taking PFS for “body weight reasons”. A prevalence of 10.2 % users of PFS for body weight reasons was observed, whose profile showed a higher proportion of: 1) females, 2) females aged 18–59, 3) individuals from Spain, 4) individuals with a BMI ≥ 25, 5) individuals with a medium education level, 6) currently employed individuals, 7) individuals who are not on a diet for overweight/obesity (72.1 % vs. 27.9 %), 8) individuals with a low level of physical activity, 9) never smokers -followed by current smokers, and of 10) individuals that consume alcohol less than once a day. The differences are only significant in cases 1), 3), 4), 7–10). As for food frequency, those who were not taking PFS for body weight reasons had a higher mean consumption of pastries/cakes and soft-drinks (times per day) as compared to those using PFS for weight control.

**Table 1:** Sample characteristics, overall and by response to question on “reasons” to take the PFS product^a^

Characteristics	All categories	Total (*n* = 2359)		Did not respond body weight (*n* = 2119, 89.8 %)		Responded body weight^c^ (*n* = 240, 10.2 %)		
		n	%	n	%	n	%	*χ* ^2^ *p*-value^*^
Gender	Males	1141	48.4	1055	49.8	86	35.8	0.000
	Females	1218	51.6	1064	50.2	154	64.2	
Age (years)	18-59	1764	74.8	1578	74.5	186	77.5	0.306
	≥60	595	25.2	541	25.5	54	22.5	
Country	Finland	401	17.0	364	17.2	37	15.4	0.000
	Germany	398	16.9	362	17.1	36	15.0	
	Italy	378	16.0	345	16.3	33	13.8	
	Romania	400	17.0	375	17.7	25	10.4	
	Spain	402	17.0	305	14.4	97	40.4	
	United Kingdom	380	16.1	368	17.4	12	5.0	
BMI (kg/m^2^)	<25	1185	50.2	1089	51.4	96	40.0	0.001
	≥25	1174	49.8	1030	48.6	144	60.0	
Education	Low	249	10.6	223	10.5	26	10.8	0.109
	Medium	1549	65.7	1379	65.1	170	70.8	
	High	561	23.8	517	24.4	44	18.3	
Employment	Currently employed	1357	57.5	1210	57.1	147	61.3	0.218
	Other groups	1002	42.5	909	42.9	93	38.7	
Dieting	Not dieting for ove/obe^b^	2247	95.2	2074	97.9	173	72.1	0.000
	Dieting for ove/obe^b^	112	4.8	45	2.1	67	27.9	
Physical activity	Low	1214	51.5	1067	50.4	147	61.3	0.006
	Moderate	1033	43.8	950	44.8	83	34.6	
	High	112	4.7	102	4.8	10	4.2	
Smoking habit	Never smoker	1100	46.6	1005	47.4	95	39.6	0.032
	Former smoker	544	23.1	488	23.0	56	23.3	
	Current smoker	715	30.3	626	29.5	89	37.1	
Alcohol consumption	0- < 1 time/day	1398	82.5	1251	82.0	147	87.5	0.074
	≥1 time/day	296	17.5	275	18.0	21	12.5	
		Mean	SD	Mean	SD	Mean	SD	*t*-test *p*-value^*^
Food frequency (times/day)	Fruit	1.34	1.04	1.35	1.06	1.25	0.94	ns
	Vegetable	0.95	0.91	0.94	0.91	1.01	0.93	ns
	Bakery and pastries	0.49	0.96	0.51	0.99	0.3	0.62	0.002
	Soft drinks	1.07	1.79	1.11	1.83	0.79	1.33	0.011
	Fast food	0.09	0.26	0.09	0.27	0.08	0.14	ns

^a^Question asked: For what reason(s)/condition(s) did you take this product? (mark all that apply) - twenty possible options were available. Variable categories: 1) did not respond “body weight”, 2) responded “body weight”; ^b^Ove/obe: overweight/obesity; ^c^Responded “body weight” among other reasons

*p < 0.05 for significance

### Country distribution of products taken for body weight reasons and of consumers dieting for overweight/obesity

**Fig. 1:**
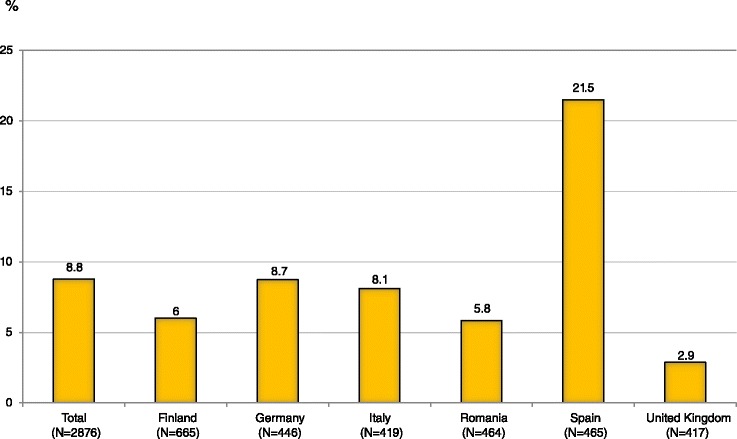
PFS products taken for “body weight reasons” (% of total PFS reported), by country

Figure 1 shows that of the total 2874 PFS products reported in the survey, 252 (8.8 %) products were being consumed for body weight reasons (by 240 PFS consumers), Spain being the country with the highest consumption of PFS for this reason (21.5 %).

**Fig. 2:**
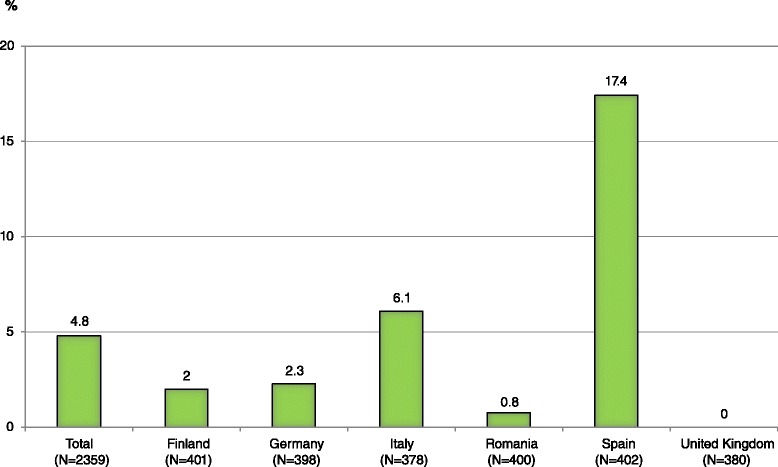
PFS Consumers “dieting for overweight/obesity” (%), by country

Figure 2 shows that of the total 2359 PFS consumers in the survey, 112 (4.8 %) reported “dieting for overweight/obesity”, Spain being the country with most dieters.

### Herbal ingredients most consumed by respondents of “body weight reasons”, “dieters for overweight/obesity” and “dieters for overweight/obesity responding body weight”

Tables 2 and 3 list the top 20 herbal ingredients consumed by each of the two groups (subsamples 1 and 2), and Table 4 lists the top 10 herbal ingredients consumed by the third group (subsample 3). *Cynara scolymus* (artichoke) is the most consumed herbal ingredient by consumers of all three groups (contained in 6.1, 7 and 8.6 % of the PFS consumed respectively) and is followed by green tea in the first group and by fennel in the other two groups.

**Table 2:** Top 20 herbal ingredients contained in the PFS taken by consumers, according to “body weight” reason^a^

PFS taken by those who responded “body weight”^b^			PFS taken by those who did not respond “body weight”^c^		
Herbal ingredients consumed	PFS (n)	% (CI 95 %)	Herbal ingredients consumed	PFS (n)	% (CI 95 %)
*Cynara scolymus* (artichoke)	72	6.1 (4.7-7.4)	*Ginkgo bilob*a (ginkgo)	191	3.1 (2.6-3.5)
*Camellia sinensis* (green tea)	37	3.1 (2.1-4.1)	*Oenothera biennis* (evening primrose)	184	2.9 (2.5-3.4)
*Foeniculum vulgare* (fennel)	34	2.9 (1.9-3.8)	*Panax ginseng* (ginseng)	160	2.6 (2.2-2.9)
*Vitis vinifera* (grapevine)	23	1.9 (1.2-2.7)	*Aloe vera* (aloe)	132	2.1 (1.8-2.5)
*Ananas comosus* (pineapple)	21	1.8 (1.0-2.5)	*Valeriana officinalis* (valerian)	123	2.0 (1.6-2.3)
*Taraxacum officinale* (dandelion)	21	1.8 (1.0-2.5)	*Cynara scolymus* (artichoke)	101	1.6 (1.3-1.9)
*Pimpinella anisum* (aniseed)	19	1.6 (0.9-2.3)	*Echinacea purpurea* (echinacea)	100	1.6 (1.3-1.9)
*Rosmarinus officinalis* (rosemary)	19	1.6 (0.9-2.3)	*Foeniculum vulgare* (fennel)	98	1.6 (1.3-1.9)
*Linum usitatissimum* (flax)	17	1.4 (0.8-2.1)	*Melissa officinalis* (lemon balm)	95	1.5 (1.2-1.8)
*Vaccinium myrtillus* (bilberry)	17	1.4 (0.8-2.1)	*Glycine max* (soy bean)	91	1.5 (1.2-1.8)
*Citrus limon* (lemon)	15	1.3 (0.6-1.9)	*Vaccinium myrtillus* (bilberry)	83	1.3 (1.0-1.6)
*Raphanus sativus* (radish)	15	1.3 (0.6-1.9)	*Echinacea angustifolia* (echinacea)	77	1.2 (1.0-1.5)
*Urtica dioica* (common nettle)	15	1.3 (0.6-1.9)	*Harpagophytum p.* (devil’s claw)	75	1.2 (0.9-1.5)
*Zingiber officinale* (ginger)	15	1.3 (0.6-1.9)	*Passiflora incarnata* (passionflower)	74	1.2 (0.9-1.5)
*Matricaria chamomilla* (Hungarian camomile)	15	1.3 (0.6-1.9)	*Zingiber officinale* (ginger)	74	1.2 (0.9-1.5)
*Malus pumila* (apple)	14	1.2 (0.6-1.8)	*Allium sativum* (garlic)	71	1.1 (0.9-1.4)
*Aloe vera* (aloe)	13	1.1 (0.5-1.7)	*Pimpinella anisum* (aniseed)	70	1.12 (0.9-1.4)
*Equisetum arvense* (horsetail)	13	1.1 (0.5-1.7)	*Glycyrrhiza glabra* (licorice)	67	1.1 (0.8-1.3)
*Fucus vesiculosus* (kelp)	13	1.1 (0.5-1.7)	*Oenothera spec (evening primrose)*	65	1.0 (0.8-1.3)
*Olea europaea* (olive tree)	13	1.1 (0.5-1.7)	*Mentha piperita* (peppermint)	64	1.0 (0.8-1.3)

^a^Question asked: *For what reason(s)/condition(s) did you take this product? (mark all that apply)* - twenty possible options were available. ^b^Those who responded “body weight” i.e. Subsample 1; at least one of the options chosen included “body weight”. ^c^Neither of the options chosen included “body weight”

**Table 3:** Top 20 herbal ingredients contained in PFS taken by consumers who “are/are not” “dieting for overweight/obesity”^a^

Responded “Dieting for ove/obe”^b^			Did not respond “Dieting for ove/obe”^c^		
Herbal ingredients consumed	PFS (n)	% (CI 95 %)	Herbal ingredients consumed	PFS (n)	% (CI 95 %)
*Cynara scolymus* (artichoke)	34	7.0 (6.4-7.7)	*Oenothera biennis* (Evening primrose)	193	2.8 (2.4-3.2)
*Foeniculum vulgare* (fennel)	17	3.5 (2.3-3.2)	*Ginkgo biloba* (ginkgo)	191	2.7 (2.3-3.1)
*Taraxacum officinale* (dandelion)	14	2.9 (2.0-2.8)	*Panax ginseng* (ginseng)	166	2.4 (2.0-2.8)
*Ananas comosus* (pineapple)	13	2.7 (1.7-2.5)	*Aloe vera* (aloe)	143	2.1 (1.7-2.4)
*Matricaria chamomilla* (Hungarian camomile)	11	2.3 (1.6-2.4)	*Cynara scolymus* (artichoke)	139	2.0 (1.7-2.3)
*Camellia sinensis* (green tea)	10	2.1 (1.4-2.1)	*Valeriana officinalis* (valerian)	120	1.7 (1.4-2.0)
*Rosmarinus officinalis* (rosemary)	10	2.1 (1.3-2.0)	*Foeniculum vulgare* (fennel)	115	1.7 (1.3-2.0)
*Fucus vesiculosus* (kelp)	9	1.9 (1.1-1.8)	*Echinacea purpurea* (echinacea)	102	1.5 (1.2-1.8)
*Paullinia cupana* (guarana)	9	1.9 (1.1-1.8)	*Melissa officinalis* (lemon balm)	102	1.5 (1.2-1.8)
*Vitis vinifera* (grapevine)	9	1.9 (1.1-1.7)	*Glycine max* (soy bean)	98	1.4 (1.1-1.7)
*Raphanus sativus* (radish)	8	1.7 (1.1-1.7)	*Vaccinium myrtillus* (bilberry)	95	1.4 (1.1-1.7)
*Sambucus nigra* (elder)	8	1.7 (0.9-1.6)	*Zingiber officinale* (ginger)	87	1.3 (1.0-1.5)
*Carica papaya* (papaya)	7	1.5 (0.9-1.5)	*Pimpinella anisum* (aniseed)	82	1.2 (0.9-1.4)
*Citrus limon* (lemon)	7	1.5 (0.8-1.4)	*Echinacea angustifolia* (Echinacea)	79	1.1 (0.9-1.4)
*Pimpinella anisum* (aniseed)	7	1.5 (0.8-1.4)	*Vitis vinifera* (grapevine)	78	1.1 (0.9-1.4)
*Silybum marianum* (milk thistle)	7	1.5 (0.8-1.4)	*Camellia sinensis* (green tea)	77	1.1 (0.9-1.4)
*Smilax officinalis* (sarsaparilla)	7	1.5 (0.8-1.4)	*Linum usitatissimum* (flax)	75	1.1 (0.8-1.3)
*Asparagus officinalis* (asparagus)	6	1.2 (0.8-1.4)	*Passiflora incarnate* (passionflower)	75	1.1 (0.8-1.3)
*Equisetum spec.* (horsetail & scouring rush)	6	1.2 (0.8-1.3)	*Harpagophytum p.* (devil’s claw)	74	1.1 (0.8-1.3)
*Cassia senna* (senna)	5	1.0 (0.8-1.3)	*Allium sativum* (garlic)	73	1.1 (0.8-1.3)

^a^Question asked: *Are you following any special diet(s) which would cause you to avoid certain foods? (mark all that apply) –* sixteen possible options were available; ^b^Subsample 2; at least one of the options chosen included “overweight/obesity”. ^c^Neither of the options chosen included “overweight/obesity”

**Table 4:** Top 10 herbal ingredients contained in PFS taken by consumers responding “body weight” while “dieting-for-overweight/obesity”^a^

Herbal ingredients consumed	PFS (n)	% (CI 95 %)
*Cynara scolymus* (artichoke)	27	8.6 (5.5-11.7)
*Foeniculum vulgare* (fennel)	13	4.1 (1.9-6.3)
*Ananas comosus* (pineapple)	11	3.5 (1.5-5.5)
*Matricaria chamomilla* (Hungarian camomile)	10	3.2 (1.2-5.1)
*Taraxacum officinale* (dandelion)	9	2.9 (1.0-4.7)
*Fucus vesiculosus* (kelp)	8	2.5 (0.8-4.3)
*Raphanus sativus* (radish)	8	2.5 (0.8-4.3)
*Rosmarinus officinalis* (rosemary)	8	2.5 (0.8-4.3)
*Camellia sinensis* (green tea)	7	2.2 (0.6-3.9)
*Carica papaya* (papaya)	7	2.2 (0.6-3.89)

^a^Subsample 3: at least one of the options chosen included “body weight^”^ and at least one of the options chosen included “dieting-for-overweight/obesity”

### BMI differences between consumers and non-consumers of the 5 most used herbal ingredients

Table 5 shows the BMI differences between consumers and non-consumers of the top 5 herbal ingredients consumed for “body weight reasons”, when using a) subsample 1 or b) the entire survey sample. In the first case, no significant differences were observed. However, in the second case a greater proportion of consumers of PFS containing artichoke (58.4 %) and green tea (63.2 %) have a BMI ≥ 25 kg/m^2^ as compared to non-consumers (49.1 and 49.7 % respectively; *p* = 0.019 and *p* = 0.043 respectively).

**Table 5:** BMI differences between consumers and non-consumers of the top 5 herbal ingredients consumed for “body weight” reasons^a^

		BMI				
		<25 kg/m^2^		≥25 kg/m^2^		
Top 5 herbal ingredients consumed for “body weight”	Consumption group	n	%	n	%	Chi^2^
						*p*-value*
a) When using only the subsample 1^b^ (*n* = 240)						
*Cynara scolymus* (artichoke)	Consumers	24	33.3	48	66.7	0.168
	Non-consumers	72	42.9	96	57.1	
*Foeniculum vulgare ssp.* (fennel)	Consumers	15	40.5	22	59.5	0.942
	Non-consumers	81	39.9	122	60.1	
*Camellia sinensis* (green tea)	Consumers	17	50	17	50	0.199
	Non-consumers	79	38.3	127	61.7	
*Vitis vinifera* (grapevine)	Consumers	6	26.1	17	73.9	0.152
	Non-consumers	90	41.5	127	58.5	
*Ananas comosus* (pineapple)	Consumers	10	47.6	11	52.4	0.456
	Non-consumers	86	39.3	133	60.7	
b) When using the entire sample of consumers (*n* = 2359)						
*Cynara scolymus* (artichoke)	Consumers	72	41.6	101	58.4	0.019
	Non-consumers	1113	50.9	1073	49.1	
*Foeniculum vulgare ssp.* (fennel)	Consumers	71	53.8	61	46.2	0.401
	Non-consumers	1114	50	1113	50	
*Camellia sinensis* (green tea)	Consumers	32	36.7	55	63.2	0.043
	Non-consumers	1142	50.3	1130	49.7	
*Vitis vinifera* (grapevine)	Consumers	43	49.4	44	50.6	0.878
	Non-consumers	1142	50.3	1130	49.7	
*Ananas comosus* (pineapple)	Consumers	21	60	14	40	0.244
	Non-consumers	1164	50.1	1160	49.9	

^a^Differences are analysed when using a) subsample 1 (respondents of “body weight”) and b) the entire survey sample; ^b^Subsample 1: respondents of “body weight”; *p < 0.05 for significance

Table 6 shows BMI differences between consumers and non-consumers of the top 5 herbal ingredients consumed by “dieters for overweight/obesity”, when using a) the subsample 2 (“dieters") or b) the entire survey sample. In the subsample, the proportion of consumers with BMI ≥ 25 is lower among consumers of *Ananas comosus (*pineapple)-containing PFS (38.5 %) as compared to non-consumers (81.8 %) (*p* = 0.000). In the full sample the proportion of consumers with BMI ≥ 25 is greater among those using artichoke-containing PFS (58.4 %) than among non-consumers (49.1 %) (*p* = 0.019).

**Table 6:** BMI differences between consumers and non-consumers of the top 5 herbal ingredients used by “dieters for overweight/obesity”^a^

		BMI				
		<25 kg/m^2^		≥25 kg/m^2^		
Top 5 herbal ingredients consumed by “dieters for overweight/obesity”	Consumption group	n	%	n	%	Chi^2^
						*p*-value*
a) When using only the subsample 2^b^ (*n* = 112)						
*Cynara scolymus* (artichoke)	Consumers	9	26.5	25	73.5	0.590
	Non-consumers	17	21.8	61	78.2	
*Foeniculum vulgare ssp.* (fennel)	Consumers	5	29.4	12	70.6	0.511
	Non-consumers	21	22.1	74	77.9	
*Taraxacum officinale* (dandelion)	Consumers	3	21.4	11	78.6	0.866
	Non-consumers	23	23.5	75	76.5	
*Ananas comosus* (pineapple)	Consumers	8	61.5	5	38.5	0.000
	Non-consumers	18	18.2	81	81.8	
*Matricaria chamomilla* (chamomile)	Consumers	1	9.1	10	90.9	0.243
	Non-consumers	25	24.8	76	75.2	
b) When using the entire sample of consumers (*n* = 2359)						
*Cynara scolymus* (artichoke)	Consumers	72	41.6	101	58.4	0.019
	Non-consumers	1113	50.9	1073	49.1	
*Foeniculum vulgare ssp.* (fennel)	Consumers	71	53.8	61	46.2	0.401
	Non-consumers	1114	50	1113	50	
*Taraxacum officinale* (dandelion)	Consumers	39	48.8	41	51.3	0.787
	Non-consumers	1146	50.3	1133	49.7	
*Ananas comosus* (pineapple)	Consumers	21	60	14	40	0.244
	Non-consumers	1164	50.1	1160	49.9	
*Matricaria chamomilla* (chamomile)	Consumers	32	47.8	35	52.2	0.681
	Non-consumers	1153	50.3	1139	49.7	

^a^Differences are analysed when using a) subsample 2 (consumers responding to be “dieting for overweight/obesity”) and b) the entire survey sample; ^b^Subsample 2: consumers responding to be “dieting for overweight/obesity”; *p < 0.05 for significance

BMI differences among consumers and non-consumers of herbal ingredients taken by the third group could not be analysed due to the small size of the sample. Only BMI differences among consumer and non-consumers of artichoke were tested and they were not significant (*p* = 0.826). Country comparisons could not be performed either due to sample size restrictions.

### Country distribution of the number of artichoke-containing PFS used for body weight and other health reasons

**Fig. 3:**
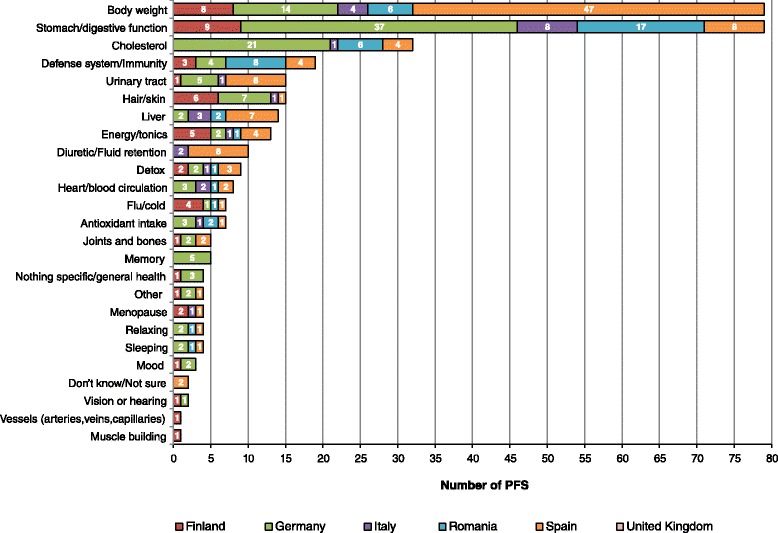
Number of *Cynara scolymus* (artichoke)-containing PFS used for body weight and other health reasons, by country

Figure 3 shows the number of artichoke-containing PFS used for body weight and other health reasons in each country. The three first reasons for taking artichoke-containing products were “body weight”, “stomach/digestive function” and “cholesterol”. Spain was the country with more PFS consumed for body weight reasons (47/79), followed by Germany (with 14/79). However, the same total number of products were used for stomach/digestive function, being most used in Germany (37/79), followed by Romania (17/79). Cholesterol is the third health reason reported by users of artichoke-containing products, being most used in Germany (21/32).

## Discussion

The analysis presented here provides an overview of the herbal ingredients contained in PFS that were used by specific consumers in six European countries, who participated in the PlantLIBRA PFS Consumer Survey 2011. The herbal ingredients are identified in those PFS consumers that a) use these products for body weight reasons, b) are overweight/obesity dieters, and c) use PFS for body weight reasons and are also dieting. The study also explores the relationship between the use/non-use of the top weight-loss PFS herbal ingredients and self-reported BMI of survey participants.

The PFS consumers who take these products for weight control are predominantly women, living in Spain, overweight and obese (with BMI ≥ 25), non-dieters, with low physical activity, never smokers, low alcohol consumers, and less frequent consumers of bakery, pastries and soft-drinks. This profile suggests that individuals who use PFS for body weight reasons are health conscious and may turn to these products with the belief that this is a safe/innocuous and effort-free strategy to lose or maintain weight, a belief that other researchers have identified [50, 51]. Other studies have reported dietary-supplement consumer profiles with similar gender results to those of the present study, but with disparate results for the other factors [9, 44, 47, 52].

The present study also found that of the total 2874 PFS products consumed, 252 (8.8 %) products were reported to be consumed for body weight reasons in the previous 12 months by 240 PFS consumers of the total sample *n* = 2359, i.e. a prevalence of weight-loss PFS users of 10.2 %. In a US study, Blanck et al. (2007) reported a prevalence of 8.7 % of past year use of “non-prescription weight-loss supplements” (including dietary supplements and natural or herbal weight loss aids not prescribed by a doctor), using data from the 2002 US National Physical Activity and Weight Loss Survey (*n* = 9,403). Of the products reported by past-year weight-loss supplement users, 73.8 % contained a stimulant including ephedra, bitter orange, caffeine, guaraná, and kola nut [44]. In a study using data on CAM use from the 2002 US National Health Interview Survey (NHIS) Alternative Medicine Supplement (*n* = 31,044), Bertisch et al. (2008) reported higher prevalence of “natural herbs use” (between approximately 17 % and just over 20 % depending on BMI category, with normal weight individuals showing the highest rate); but this study focused on CAM therapies use and did not specify the format in which the natural herbs were used [45]. Another survey of US adults, a computer-assisted telephone interview conducted by the Center for Survey Research and Analysis at the University of Connecticut in 2005–2006, reported a much higher prevalence of use: of the adults who made a serious weight-loss attempt (*n* = 1,444), 33.9 % reported ever having used a “dietary supplement for weight loss” (including “over-the-counter appetite suppressants, herbal products, or weight-loss supplements”, although not distinguishing between them) [9]. Lastly, in their recent study (*n* = 2,732) in the city of Pelotas, Brazil, Machado et al. (2012) reported that the prevalence of use of “substances for weight-loss” was 12.8 %; however, these substances included teas, dietary supplements (unspecified) and medicines [47]. It is important to mention that all these studies were conducted in general populations, as opposed to PFS consumers.

A comparison of results among the scarce publications evaluating the use of weight-loss supplements at the population level is limited. The studies varied in the terminology used (concepts and definitions ranged between “natural herbs”, “non-prescription weight loss supplements”, or “substances for weight-loss”), study designs, sample sizes, and data collection methodology. The present study is the first study to evaluate the use of herbal weight-loss supplements in consumers of PFS in six European countries, having harmonised the terminology and methods used across countries.

The present study estimated the prevalence of dieting for overweight/obesity in the PlantLIBRA Survey of PFS consumers [43]: 4.8 % (*n* = 113) of 2359 PFS consumers in the six European countries. Similar rates were reported in one study [44], where 4.4 % of those currently trying to maintain the same weight were users of weight-loss dietary supplements during the past year; however, 16.1 % of those currently trying to lose weight reported past-year use of these products (around a four-fold higher rate). In addition, Pillitteri et al. (2008) observed much higher rates, reporting that of the adults who made a serious weight-loss attempt (*n* = 1,444), 33.9 % had used a dietary supplement for weight loss [9]. These findings are similar to those of Machado et al. (2012), who reported a prevalence of 48.4 % for use of weight loss supplements in those who tried to lose weight [47]. Again, comparisons between studies are difficult because of study limitations in terms of design, terms used and data collection procedures.

This is the first study in a sample of PFS consumers from six EU countries that has identified the herbal ingredients contained in products used by “consumers for weight control”, by “overweight/obese dieters” and by “overweight/obese consumers who are simultaneously consuming PFS for weight control and dieting for weight control”. Artichoke was the herbal ingredient that appeared in the greatest number of PFS consumed in all three groups (6.1, 7 and 8.6 % respectively); however, these results might be driven by the high use of these artichoke-containing products reported in Spain and Germany (see the discussion further down). In addition, green tea (3.1 %) and fennel (2.9 %) were second and third in the first group. Fennel (3.5 %) and dandelion (2.9 %) were second and third in the second group. Lastly, fennel (4.1 %) and pineapple (3.5 %) were second and third in the third group. To our knowledge, only one recent US study has reported the actual herbal ingredients contained in weight-loss supplements and the prevalence of users [44]. They reported different herbal ingredients, with almost 74 % using a product classified as a stimulant, more than half (55 %) consuming product containing *Ephedra sinica* (ephedra or ma huang), one in 15 used a product containing *Citrus aurantium* (bitter orange), and one in 10 took *Garcinia cambogia* (hydroxycitric acid); other active herbal ingredients, such as conjugated linoleic acid and *Ilex paraguariensis* (yerba mate), were in very few of the products reported in the study [44].

Some literature on the effectiveness of artichoke for weight loss reveals that the scientific evidence is “insufficient to guarantee the efficacy and safety for treating obesity but could be useful to treat some of its comorbidities (i.e. hyperlipidemia)” [53]. In their review, de Villar et al. (2003) reported that it is frequently used in slimming products and as a diuretic [53]. According to the recent “Assessment report on *Cynara scolymus* L., folium”, by the European Medicines Agency (2011), other indications of traditional use (which is how it is used in Spain) include arteriosclerosis and hyperlipidemia [54]. The same report also states that “the antioxidative, hepatoprotective and choleretic effects of artichoke leaf extracts as well as lipid-lowering and anti-atherogenic activity with increased elimination of cholesterol and inhibition of hepatocellular de novo cholesterol biosynthesis have been demonstrated in various in vitro and in vivo test systems [54].

Only one publication included pineapple as an ingredient of popularly consumed weight loss products, in Spain [53]. The authors outlined the main therapeutic indications/recommendations of pineapple at that time (2003), distinguishing the “true” ones (burns, skin lesions) from the “traditional-use” ones (dyspepsia, arthralgia, arthritis, stomatitis, cellulitis, exocrine pancreatic insufficiency and obesity; including a comment of “mild diuretic effect”), and concluded that scientific evidence for weight-loss effectiveness is “untested/non-existent” [53]. However, in a very recent publication [55], the authors concluded that there might be an effect at cell level, which may be a potent modulator of obesity.

Finally, no publication was found including fennel as an ingredient of weight loss supplements, despite the extensive and recent scientific literature describing its uses and properties [56, 57]. A hypothesis for the high prevalence of consumption by our consumers using PFS for weight control and dieters might involve the fact that some of the properties attributed to fennel are to “improve digestion”, “prevent bloating” and as “flavour corrector” i.e. it might be accompanying other substances in weight-loss multi-ingredient supplements to improve digestion, neutralize intestinal gas formation and enhance their flavour [58]. Moreover, like for pineapple, advertisements promoting fennel-containing products as a slimming aid on the Internet are numerous, which may provide an additional explanation.

In Spain, the country with the highest prevalence of “body weight reason respondents” (21.5 %) and “dieters” (17.4 %) and where artichoke-containing products were most used for body weight reasons (47/79 PFS), results are consistent with the traditional use of artichoke as adjuvant of weight loss treatments, to allow a fat diet in the treatment of mild to moderate hyperlipidaemia (for reducing cholesterol) [54]. These results are also in line with some reports in the literature, such as the “White Book of herbal shops and medicinal plants”, a report about the situation of the Spanish herbal shop sector [59], in which the authors report that the top-selling products are food supplements (29 %) followed by weight control products (28 %). We explored other reasons for the use of artichoke in the six survey countries and there is agreement with the recommendations of use for stomach/digestive function and cholesterol (highest in Germany) (Fig. 3). In Germany, artichoke is used in traditional herbal medicinal products used to promote digestion (against dyspepsia, digestive complaints) [54]. Moreover, artichoke has been used in traditional medicine for centuries all over Europe as a specific liver and gallbladder remedy and several herbal drugs based on the plant are used as well for high cholesterol and digestive and liver disorders [54]. Other uses around the world include treatment for dyspepsia and chronic albuminuria [54]. We cannot know at this stage the health reasons behind the different prevalence of consumption of the same herbal ingredient across the six countries involved in our study, because of the low consumption levels observed in the different sample groups. In order to be able to discriminate more easily, we would need to have a higher concentration of consumers of a single product containing a particular herbal ingredient consumed for a single health condition. We could hypothesize that these differences may result from different regulatory restrictions between the countries (i.e. the same herbal ingredient might be used in PFS or in herbal medicinal products), market consumption trends, marketing strategies related to traditional/cultural beliefs, etc. However, further research is required to prove these hypotheses, involving a long-term prospective study design, a larger sample size, market, regulatory, and anthropological data, as well as, stratification by gender, season of the year, to name a few explanatory variables.

Our results show that, when the entire survey sample was used (*n* = 2359) to increase the power of the comparison (Tables 5 and 6), significant BMI differences were observed between consumers and non-consumers of artichoke. Also in this entire sample, differences were observed in BMI between consumers and non-consumers of green tea (third most consumed herbal ingredient of respondents of “body weight reasons”). In both cases, more consumers than non-consumers of each herbal ingredient were overweight/obese (BMI ≥ 25 kg/m^2^). Again, even though we could not analyse the products consumed in each country, and considering the high use of artichoke in the Spanish sample (Fig. 3), we hypothesized that the Spanish data could be influencing these differences observed through the Chi-square analysis. In order to further try and clarify this hypothesis, we performed lineal Spearman’s correlation analyses (not shown) using all 3 samples (entire survey, “respondents of body weight”, and “dieters for overweight/obesity”) between the variables of “consumption/non-consumption of the herbal ingredients of the most consumed PFS in each sample” and “BMI”; BMI was first included as a continuous variable, then as a dichotomous variable (BMI < 25; ≥25 kg/m^2^) and lastly, as a categorical variable of 3 categories (BMI < 25; ≥25-30; >30 kg/m^2^). Only the following two results yielded in these analyses were significant for products containing artichoke: 1) with BMI continuous, consumers of products containing artichoke tended to have a higher BMI (coefficient = 0.070, significance = 0.001); 2) with BMI dichotomous, consumers of products containing artichoke tended to be in the highest BMI range (coefficient = 0.048, significance = 0.019). These results show that, although significant, the correlations were not very strong (not very close to 1). This could indicate that the Spanish data was not influencing the global results as far as artichoke was concerned in the entire survey sample.

As for the “dieters” subsample only (*n* = 112), results presented in Table 6 show very significant BMI differences for products containing *Ananas comosus* (pineapple), with consumers having higher rates of normal weight (BMI < 25 kg/m^2^) than non-consumers. These results were in line with those observed later in the correlation analyses (not shown), which yielded the following significant results: with all 1) BMI continuous, 2) BMI dichotomous, and 3) BMI categorical, consumers of products containing pineapple tended to be in the lowest ranges of BMI, i.e. 1) coefficient = -0.250, significance = 0.008 2) coefficient = -0.329, significance = 0.000, and 3) coefficient = -0.324, significance = 0.000, respectively). These correlation results were very significant (significance < 0.01) and also stronger (closer to -1) than those for artichoke.

Finally, we took a step further and, only using the Spanish data, we performed some Chi-square tests to analyze differences in the relationship between dichotomous BMI and the consumption/non-consumption of the 5 herbal ingredients included in Tables 5 (respondents of “body weight”) and 6 (“dieters”) (not shown). The differences observed were only significant for pineapple in both subsamples, i.e. again, a higher percentage of consumers of products containing pineapple had a BMI < 25 kg/m^2^, but to a further extent in “dieters” vs. “respondents of body weight” (*p* = 0.000 vs. *p* = 0.012, respectively). These results concerning the consumption of pineapple-containing PFS by the “dieters” subsample could suggest an influence of the Spanish data on the global results.

Summarising, pineapple contained in products consumed by “dieters” show the strongest relationship with BMI, with those declaring to consume them tending to have a lower BMI or tending to belong to the lowest BMI range. The global results observed for this relationship are possibly influenced by its higher consumption in Spain. However, we do not know why this is happening or if there is an association influenced by other factors, and we cannot infer causality from these results due to the cross-sectional nature of the survey. Bertisch et al. (2008), who analysed the relationship between obesity and the use of CAM (including natural herbs), reported that adults with obesity had similar prevalence of use of natural herbs compared to normal-weight individuals, and after adjustment by some factors they were generally less likely to use most individual CAM modalities [45]. Nevertheless, Bertisch et al.’s study and our study are not comparable because they evaluated the overall use of natural herbs as a CAM modality in the general population, instead of the use of herbal ingredients among PFS consumers. To our knowledge, our study is the first study that has tested BMI differences between consumers and non-consumers of particular herbal ingredients contained in PFS.

The present study has several limitations. The survey was not designed to assess weight loss. All data were self-reported, allowing the possibility of misreporting -although with regards to the products, the interviewers verified the packaging of approximately 50 % of them. There exists the possibility of misclassification of a product as a PFS when it might be in fact an herbal medicinal product, due to the unawareness by the consumer of the legal status of the product or by a post-data-collection change of status of the product. In addition, the survey did not collect composition/label data (mostly unavailable), therefore, dosages of herbal ingredients could not be calculated for BMI/dosage analyses. The definition of the product “plant food supplement” is so specific that results can really only be compared with results from other studies with this definition. The cross-sectional nature of the survey does not allow inference of causality. The design of the survey (only including PFS consumers and quota sampling) does not allow either the weighting of the data, the extrapolation of results to the general population or the comparison with general population studies. Finally, the survey had a small sample size that allowed limited stratification and no regression analyses for assessing the association between BMI and herbal ingredients consumption vs. non-consumption and identifying significant predictors.

This study has some unique strengths. It is the first study that has identified the herbal ingredients most consumed by PFS consumers from six European countries who reported taking these products for reasons of “body weight” or who were “dieting for overweight/obesity”. In addition, the “PFS product” was very clearly defined and differentiated from other herbal products, which will allow direct comparison with future studies on weight loss and PFS consumption that might be conducted. Finally, the study has identified some of the many possibilities for future research to try and explain the differences in the use of weight-loss herbal supplements across national markets within the EU. This would encourage, for example, further research into the many aspects by which the different types of herbal products used in weight-loss/control can be differentiated, ideally using purposely collected data at the national and/or European levels.

## Conclusions

A comparison of results among the limited publications evaluating the use of weight-loss supplements at the population level is limited. Nevertheless every hint is important in finding out which are the self-treatment strategies used by overweight/obese individuals in European countries. Although limited by a small sample size, our study represents a first attempt at analysing such data in six EU countries. Our findings should encourage the conduction of further studies on this topic, including long-term and large sample-sized studies, ideally conducted in the general population. These studies would include, for example, prospective/cohort studies collecting detailed data on ingredients amounts/dosages, and identifying patterns and reasons of consumption for determining health outcomes (such as obesity); or studies interlinking data with national markets of botanical products (including weight-loss products); or regional/national nutritional/health/CAM-use surveys collecting data on the consumption of botanical products (including weight-loss ones); or national consumer surveys and health knowledge/perception surveys collecting data on these products. This additional information would help elucidate the many unknowns about the marketing, consumption and effectiveness of PFS, in particular, those specifically used as a strategy for body weight control.

## Abbreviations

BMI, body mass index; CAM, complementary and alternative medicine; PFS, plant food supplements; RCTs, randomised controlled trials
